# Effect of CeO_2_–GO Nanocomposite on the Anticorrosion Properties of Epoxy Coating in Simulated Acid Rain Solution

**DOI:** 10.3390/polym14173573

**Published:** 2022-08-30

**Authors:** Ruidan Liu, Xiaoyan Liu, Heng Yang, Handuo Jie, Tianyu Li, Kai Lyu, Surendra P. Shah

**Affiliations:** 1 College of Mechanics and Materials, Hohai University, Nanjing 210098, China; 2 Institute of Corrosion Protection, Hohai University, Nanjing 210098, China; 3 Materials & Structural Engineering Department, Nanjing Hydraulic Research Institute, Nanjing 210024, China; 4 TSMC Nanjing Co., Ltd., Nanjing 211806, China; 5 College of Water Conservancy and Hydropower Engineering, Hohai University, Nanjing 210098, China; 6 College of Civil and Transportation Engineering, Hohai University, Nanjing 210098, China; 7 College of Engineering, University of Texas at Arlington, 701 S. Nedderman Drive, Arlington, TX 76019, USA

**Keywords:** CeO_2_, nanocomposite, electrochemistry, EIS, acid rain

## Abstract

The lamellar structure of graphene oxide and the filling effect of nano-cerium oxide particles together provide a good barrier and stability to coating. In this paper, cerium oxide-graphene oxide (4:1) nanocomposite was prepared by the hydrothermal synthesis method. The effect of cerium oxide–graphene oxide (4:1) nanocomposite on the anticorrosion properties of epoxy coating in simulated acid rain solution was studied by open circuit potential (OCP), electrochemical impedance spectroscopy (EIS), Mott–Schottky curve, Tafel curve, and micromorphological characterization, in order to compare it with pure epoxy coating, graphene oxide epoxy coating, and cerium oxide epoxy coating. The obtained results showed that cerium oxide–graphene oxide (4:1) epoxy coating’s protection efficiency was as high as 98.62%. These results indicated that cerium oxide–graphene oxide modified anticorrosive coating had an excellent application prospect in an acid rain environment. Meanwhile, owing to the poor protection ability of epoxy resin and unstably hydrolysis product of CeO_2_ to the acidic medium, the resistance of CeO_2_–GO (4:1)/EP coating to acidic corrosive medium was relatively poorer than that of neutral and saline-alkali corrosive medium.

## 1. Introduction

With the continuous development of human civilization, metal materials are widely used in different environments, such as the construction industry, the shipbuilding industry, and the machinery manufacturing industry. Despite the excellent properties of metal materials, the corrosion is an inevitable challenge during its service life, which is bound to result in the deterioration of the macro-performance of metal-based infrastructures. According to the previous research, global metal corrosion costs $2.5 trillion annually, or 3.4% of global annual gross domestic product (GDP) [1,2]. This process of the rapid development of world economic construction was closely dependent on traditional fossil energy: oil, kerosene, and natural gas, accompanied by considerable emissions of pollutant gas, including SO_2_, NO_2_, carbon oxides, and sulfides. Pollutant gas can form corrosive medium in rainy and snowy weather–acid rain [3], which is considered a global environmental problem. It is widely accepted that acid rain not only causes acidification of surface waters and disrupts normal metabolic and reproductive systems, but also erodes concrete buildings and metal materials [4,5,6]. Basically, rainwater just provides the required electrolytes for the corrosion of metals, and the sedimentation of rainwater also had a scouring and damaging effect on metals, forming the electrochemical dissolution and scouring interaction between the corrosion mechanisms of metals by rainwater [7]. 

Commonly used metal anti-corrosion methods include corrosion-resistant alloys, anti-corrosion coatings, corrosion inhibitor protection, electrochemical protection, and other methods [1]. However, overall, considering the protection efficiency and the economic costs, the anti-corrosion coating was the most widely used way to improve the anti-corrosion resistance of the materials [8,9,10,11]. Essentially, the coating can act as a physical barrier against corrosive media, which will protect the metal materials from the corrosion ions. Anti-corrosion coatings are widely used in acid rain and acid environments. Li et al. [12] investigated the corrosion resistance of an Ni-P coating on A3 steel in an acid rain environment, and found that the corrosion resistance of the coating was better than that of 1Cr18Ni9Ti stainless steel. Liu et al. [13] prepared a graphene anti-corrosion coating with a silane coupling agent and found that the coating had good corrosion resistance to salt spray. Nagai et al. [14] studied the corrosion resistance of diamond-like carbon (DLC) coatings in hydrochloric, nitric, and sulfuric acid solutions, and the experimental results proved that DLC coatings reduced the elution of metal ions in each acid solution by more than 90 percent. Wang et al. [15] developed a pH-reactive epoxy resin composite coating that would release nano-benzotriazole to display good anti-corrosion and active repair functions. Owing to metal cations being extremely active in an acidic environment, the addition of the nano material modification method can show a better protective effect.

Graphene had a unique two-dimensional structure, with a high specific surface area, high strength, toughness, good chemical stability, impermeability to most molecules, and excellent conductivity. Graphene oxide (GO) had good compatibility, which means it can be coated onto the metal surface in an easier way. GO can significantly improve the corrosion resistance of substrate materials and is widely used in anti-corrosion and wear-resistant coatings [16,17,18,19]. Ryu et al. [20] proposed electrophoretic deposition (EPD) to coat GO with varying sizes on the surface of steel components and found that the corrosion resistance of steel components increased with the size of GO. Verma et al. [21] used graphene nanosheets to enhance the Al–Ti coating, while the seawater wear rate of the coating decreased from 11 × 10^−14^ m^3^/Nm to 4 × 10^−14^ m^3^/Nm. Fan et al. [22] introduced an amino group on the surface of graphene to obtain a modified graphene oxide composite coating, and the coating containing modified graphene oxide was 10 times more corrosive than the coating without it. Geng et al. [23] developed a graphene-oxide-modified silane composite coating with a thickness of 45 μm to perform good corrosion resistance, and the maximum protection efficiency under chloride ion medium reached 99.36%. 

Owing to its inherent hydroxyl groups, nano-oxides such as SiO_2_ and TiO_2_ can be attached to the surface of GO for surface modification. The increase in oxygen-containing functional groups on GO makes it more reactive than graphene and can improve its properties through various reactions with oxygen-containing functional groups. The hybrid material with a coupling effect can be uniformly dispersed in the coating to improve the interface bonding strength [5,24,25]. Rare earth elements as corrosion inhibitors have a positive adjustment effect on the coating structure [26,27,28,29]. Cerium oxide (CeO_2_) is a typical rare earth element oxide. The coating doped with nanometer CeO_2_ has better corrosion resistance and penetration resistance [30]. Wu et al. [31] discussed that the adsorption of CeO_2_ on the surface of low-carbon steel can form a passivation layer and further inhibit the corrosion of the metal. Li et al. [32] prepared a graphite/CeO_2_-reinforced Co-based coating to study the synergistic effect of graphite and CeO_2_, which led to an improvement in the microhardness, wear resistance, and corrosion resistance of the coating. You et al. [33] studied the NiCo-CeO_2_ composite coating with excellent corrosion resistance when the nano-CeO_2_ particle concentration was 10 g/L. The polarization resistance of the graphene-p-aminobenzoic acid-CeO_2_ coating prepared by Li et al. [34] was four times higher than that of the blank coating, and the corrosion resistance was greatly improved. An et al. [35] investigated 1 wt% CeO_2_/epoxy and VTEO-CeO_2_/epoxy coatings, finding that they had the highest system resistance value of 2.96 × 10^5^ ohm⋅cm^2^ after being exposed to 3.5 wt% NaCl solution for 240 h. Liu et al. [36] studied CeO_2_–GO nanocomposites with different CeO_2_ to GO mass ratios by the hydrothermal synthesis method and indicated that the CeO_2_–GO (4:1) nanocomposite had excellent anticorrosion properties and eliminated the agglomeration of graphene oxide to some extent. It also analyzed CeO_2_–GO (4:1) epoxy coating had excellent anticorrosion properties in seawater and saline–alkali environments [37,38].

This work investigated the effect of the preparing the CeO_2_–GO (4:1) nanocomposite by the hydrothermal synthesis method on the anticorrosion properties of epoxy coating in simulated acid rain solution. The corrosion situation was observed by open circuit potential (OCP), electrochemical impedance spectroscopy (EIS), Mott–Schottky curve, Tafel curve, and stereo microscope. The effect of time extension on the corrosion resistance was also evaluated and the results indicated that the CeO_2_–GO (4:1)/EP coating had excellent barrier performance to the erosive medium. At the same time, the accumulation of corrosion products and the good compactness of the coating limited the further penetration of erosive ions and slowed the corrosion rate. The results of this work are expected to enhance the application of cerium oxide-modified graphene oxide anticorrosive coating in an acidic engineering environment.

## 2. Materials and Methods

### 2.1. Raw Materials

In this study, graphene oxide (SE2430W) was purchased from Changzhou Sixth Element Materials Technology Co., Ltd., Changzhou, China. The basic performance parameters of graphene oxide were obtained experimentally, as listed in Table 1. The epoxy (WSR6101 E-44) and epoxy AB glue were supplied by Nantong Xingchen Synthetic Material Co., Ltd., Nantong, China. The cerium hexahydrate nitrate ((CeNO_3_)_3_·6H_2_O) was an analytical reagent obtained from Shanghai Aladdin Bio-Chem Technology Co., Ltd., Shanghai, China. The acetone (CH_3_COCH_3_), ammonium hydroxide, sodium sulfate (NaSO_4_), and nitric acid (HNO_3_) were supplied by Chengdu Colon Chemicals Co., Ltd., Chengdu, China. In the experiment, Q235 carbon steel with a height of 5 mm and a diameter of 10 mm was used to evaluate the anticorrosion performance of the developed coatings. Table 2 shows the composition of Q235 carbon steel.

### 2.2. Preparation of CeO_2_–GO (4:1) Nanocomposite Epoxy Coating

**Figure 1:**
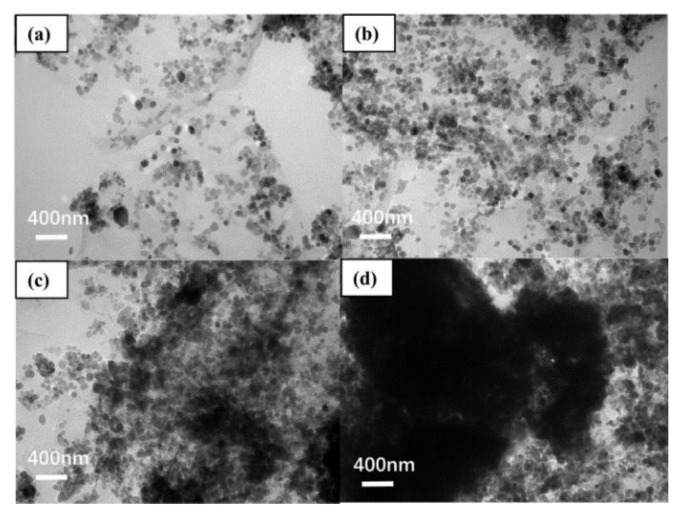
Transmission electron micrographs: (**a**) CeO_2_–GO (2:1); (**b**) CeO_2_–GO (4:1); (**c**) CeO_2_–GO (6:1); (**d**) CeO_2_–GO (8:1) [36].

CeO_2_–GO (4:1) nanocomposites were prepared using a hydrothermal synthesis method [39]. Previous research has illustrated that the CeO_2_–GO (4:1) nanocomposite eliminated the agglomeration of graphene and prepared a graphene epoxy coating with good dispersion. Figure 1 shows the micromorphological features of CeO_2_–GO nanocomposites. Above all, in order to obtain the thin-flake GO, a certain amount of GO slurry was weighed and dissolved in dialyzed water. Afterwards, the thin-flake GO was stripped by an ultrasonic cell crusher(Shanghai Jingxin Industrial Development Co., Ltd., Shanghai, China). After this, an appropriate amount of cerium nitrate hexahydrate was mixed with the GO solution, followed by ultrasonic stirring and magnetic stirring for 30 min, respectively. Ammonium hydroxide was then added after the solution was stirred for an adequate dispersion condition. Next, the obtained solution was placed into a high-pressure reaction kettle and reacted for 24 h at 180 °C. Finally, the solution was washed and filtered by deionized water and anhydrous ethanol, respectively, before extracting the solid phase in the solution. The extracted solid phase was dried and grounded to obtain CeO_2_–GO nanocomposites. According to the above process, the CeO_2_–GO nanocomposite with a mass ratio of CeO_2_/GO = 4:1 was prepared for further manufacturing of CeO_2_–GO (4:1) epoxy coating.

**Figure 2:**
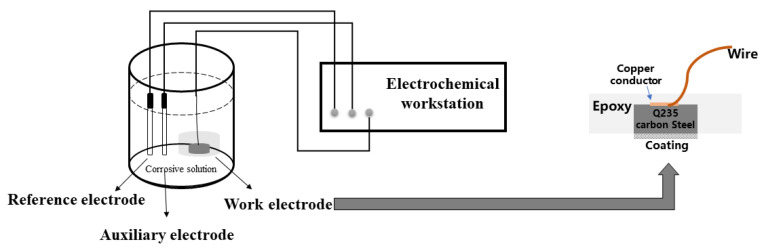
The coating’s sample diagram.

The cylindrical Q235 carbon steel was used for inspection work to compare the anti-corrosion performance of each coating. The steps for preparing the sample were as follows. Initially, the copper conductor was fixed on one surface of the steel sample, so that the unconnected copper conductor had one side down. Then, the steel was placed in the center of a cylindrical plastic mold with 20 mm in diameter and 15 mm in height. Next, the AB epoxy was allowed to fill the entire mold until it covered the top surface of the steel sample, and the mixture remained in the mold for 24 h in order to fully harden the epoxy. After the sample was demolded, in order to expose the steel sample on the bottom surface and obtain a relatively smooth bottom surface to improve the direct connection between the coating and the steel sample, it is necessary to remove the excess AB epoxy resin, so the bottom surface of the sample was grinded and polished sequentially with 400-, 800-, 1000-, 1200-, and 2000-mesh abrasive papers. CeO_2_–GO (4:1), 0.5% by mass of epoxy resin (by mass), was added to 4 g of epoxy resin after a 30 min ultrasonic dispersion process. After the above, in order to improve the mixture even futher, the mixture was heated with magnetic stirring for 30 min, and the acetone in the mixture could be evaporated. The prepared mixture was coated on the bottom surface of Q235 steel with a coating machine, and the coating thickness was controlled to be 100 μm by a thickness gauge Finally, the CeO_2_–GO (4:1)/EP composite coating was obtained after being cured at room temperature for 24 h. The CeO_2_/EP coating and GO/EP coating were prepared in a similar manner for comparison. Figure 2 shows the coating’s sample diagram.

### 2.3. Preparation of Simulated Acid Rain Solution

In the acid rain, SO_4_^2−^ and NO_3_^−^ are the main acid-causing substances, which are converted from sulfur oxides (SO_2_) and nitrogen oxides (NO, NO_2_). The pH of the simulated acid rain solution was set to be 2.3 in this research. In order to prepare simulated sulfuric acid rain and nitric acid rain, Na_2_SO_4_ solution with a concentration of 1 g/L was prepared, and its pH was adjusted to 2.3 with HNO_3_.

### 2.4. Testing Procedures

#### 2.4.1. Micromorphological Characterization

HIROX-KH7700 stereomicroscope (Shanghai HIROX Instrument Technology Co., Ltd., Shanghai, China) was used to observe the corrosion condition of the substrate coated with each coating. Three-dimensional morphologies of corroded parts on metal substrates were fitted. The corroded areas of metal substrates were analyzed and calculated by contour maps of different depths, as well as the corrosion depth.

#### 2.4.2. Electrochemical Testing

The electrochemical workstation of PARSTAT 2273 type (AMETEK Group of Companies, San Diego, CA, USA) was used for electrochemical testing. The main test methods included open circuit potential test (OCP), electrochemical impedance spectroscopy (EIS), Tafel curve test, and Mott–Schottky curve test. The scanning range of open circuit potential (OCP) was −200 mV~1200 mV. The frequency range of EIS was 10^5^~10^−2^ Hz, and the amplitude was 10 mV. The scanning range of the potentiometric polarization curve was −300~300 mV, and the scanning rate was 1 m V/s. The adopted Mott–Schottky frequency was 1000 Hz, and the scanning interval was −1~0.5 V. Previous research indicated excessive amount of CeO_2_ particle would gradually agglomerate together by the GO tablets, leading to poor dispersion performance, while a minor amount of CeO_2_ particle could not improve the aggregation of GO effectively to show good anticorrosion. CeO_2_–GO (4:1) nanocomposites eliminated the agglomeration of graphene to some extent, as well as the graphene, and further promoted the coating’s anti-corrosion performance. The samples of EP coating, GO/EP coating, CeO_2_/EP coating, and CeO_2_–GO (4:1)/EP coating, denoted as C1, C2, C3, and C4, respectively, were tested and compared.

## 3. Anti-Corrosion Performance

### 3.1. Open Circuit Potential (OCP) Analysis

**Figure 3:**
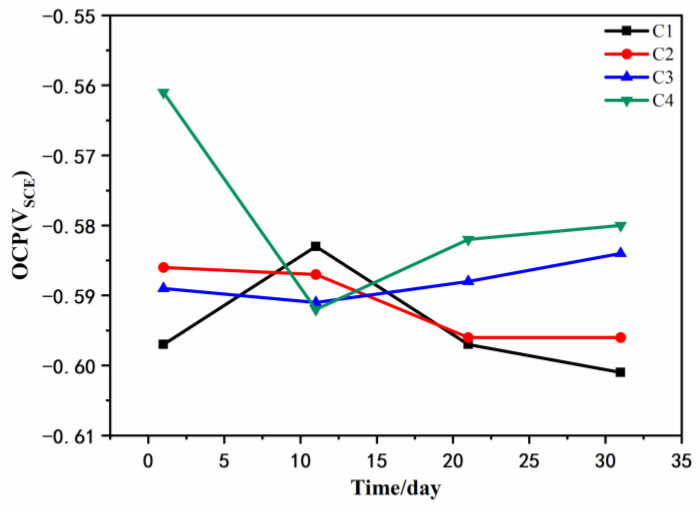
Open circuit potential of coatings after immersion.

Figure 3 shows the open circuit potential for C1, C2, C3, and C4 after being immersed in the simulated acid rain solution for 1 d, 11 d, 21 d, and 31 d. It can be seen from Figure 3 that the open circuit potential of C4 was the largest, which was −580 mV after immersion for 31 d, while the open circuit potential of C3 was −584 mV, that of C2 was −596 mV, and that of C1 was −601 mV. Generally, the numerical value of the open circuit potential represents the magnitude of corrosion tendency [40] and, the smaller the open circuit potential, the lower corrosion tendency. Thus, C4 has the smallest corrosion tendency. When considering its time-dependent performance, resulting from the penetration of the corrosive medium, the protective performance of the coating decreases and the open circuit potential decreases. The open circuit potentials of the coatings all tended to decrease first and then increase. Considering that the corrosive solution is acidic with a pH of 2.3, it has strong erosiveness to the coating, with the open circuit potentials decreased. When the metal oxides are adsorbed on the surface of the metal substrate, the further development of corrosion is delayed, so the open circuit potential tends to decrease and be stable. When corrosion begins to occur, C1 is very poor in anticorrosion and the rebar corrodes severely. Owing to the accumulation of corrosion products, the open circuit potential of C1 was improved at 10 d, and then seriously failed.

The open circuit potentials of the four coatings showed little difference after being immersed for 31 d. When the pH of the solution is greater than 5, the corrosion of the metal substrate is mainly oxygen absorption corrosion. However, in severe acid rain environments (pH < 4.7), the main corrosion is hydrogen evolution corrosion. At the same time, the acid corrosive medium corrodes the metal substrate more severely than the neutral and alkaline corrosive medium, and the substrate undergoes hydrogen evolution corrosion and generates Fe^2+^ at a faster rate. The open circuit potential showed that the corrosion tendency of the four coatings was C1 > C2 > C3 > C4.

### 3.2. Electrochemical Alternating Current Impedance Spectroscopy (EIS) Analysis

**Figure 4:**
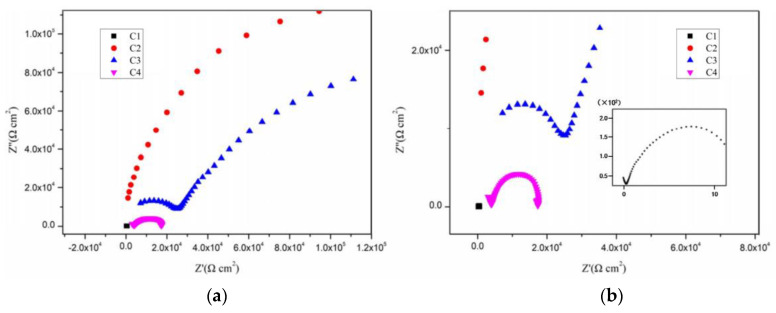
Nyquist plots of coatings after immersion for 1 d: (**a**) Nyquist patterns for 1 d and (**b**) enlarged view of the high frequency of Nyquist patterns.

**Figure 5:**
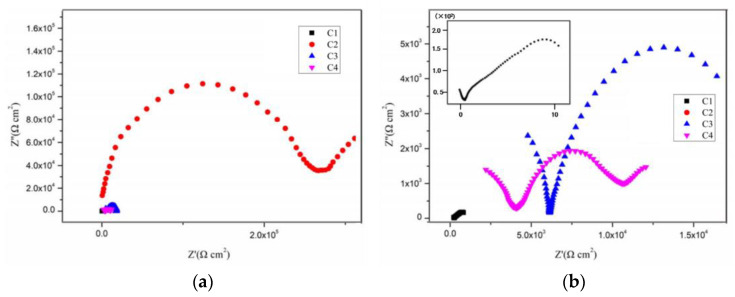
Nyquist plots of coatings after immersion for 11 d: (**a**) Nyquist patterns for 11 d and (**b**) enlarged view of the high frequency of Nyquist patterns.

**Figure 6:**
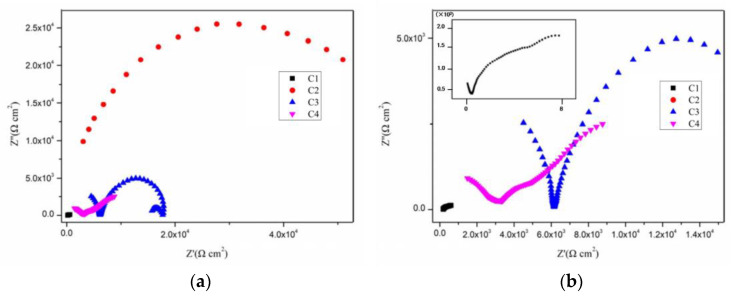
Nyquist plots of coatings after immersion for 21 d: (**a**) Nyquist patterns for 21 d and (**b**) enlarged view of the high frequency of Nyquist patterns.

**Figure 7:**
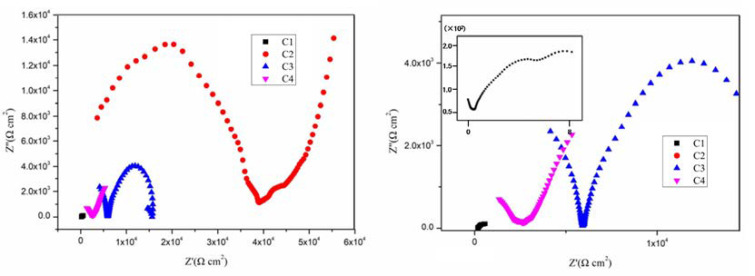
Nyquist plots of coatings after immersion for 31 d: (**a**) Nyquist patterns for 31 d and (**b**) enlarged view of the high frequency of Nyquist patterns.

Figure 4, Figure 5, Figure 6 and Figure 7 show the Nyquist patterns of carbon steel coated with C1, C2, C3, and C4 immersed in the simulate acid rain solution for 1 d, 11 d, 21 d, and 31 d. As can be seen from Figure 4, the capacitive arc radius of C4 was significantly larger than that of the other three coatings after soaking for 1 d. The results indicated that C4 had the largest resistance value and the best anticorrosion performance. However, by observing the curves of C1, C2, and C3, it was found that incomplete capacitive reactance arcs appeared in the high frequency region, caused by the phase shift of high frequency capacitive reactance. The appearance of such a high-frequency incomplete semi-circle could not truly reflect the surface state of the coating. The reason for this phenomenon is mainly the poor barrier and shielding performance of the coating. Thus, its impedance is small. Meanwhile, the species and concentration of ions in the simulated acid soaking solution are lower than those in the saline-alkali solution, and the migration rate of the ions decreases, resulting in the conductivity of the solution [38]. In addition, owing to the existence of the internal resistance of the reference electrode, the abovementioned three factors work together to cause a high-frequency capacitive phase shift in the impedance spectrum. The high-frequency phase shift end point can reflect the performance of the coating to a certain extent. The higher the end point of the high-frequency phase shift, the higher the impedance modulus of the starting point.

By comparison, it can be found that the impedance modulus value at the end point of the high-frequency phase shift of the three is C3 > C2 > C1. With the prolongation of immersion time, the capacitive arc radius of each coating decreases continuously and the high-frequency phase shift point keeps moving to the left, indicating that the erosive ions continue to invade the coating. The capacitive arc radius of C4 maintained a high level, which indicates excellent anticorrosion performance. It can be seen from Figure 4, Figure 5, Figure 6 and Figure 7 that C4 exhibited a high-frequency capacitive resistivity phase shift for the first time after immersion for 31 days and its impedance value was always large, while the capacitive reactance arc and high-frequency phase shift point of the other three coatings were constantly decreasing, showing the other three coatings’ poor resistance to acidic aggressive media.

**Figure 8:**
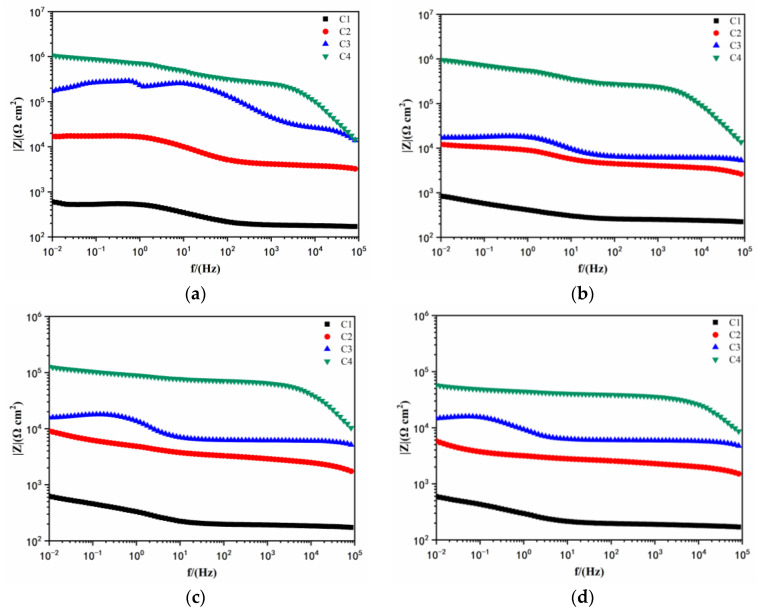
Bode pattern after immersion: (**a**) 1 d; (**b**) 11 d; (**c**) 21 d; and (**d**) 31 d.

Figure 8 shows the Bode pattern of each coating immersed for 1 d, 11 d, 21 d, and 31 d, respectively. It can be seen that, when the immersion time is 1 d, the low-frequency impedance modulus of C4 was 1.068 × 10^6^ Ω cm^2^, while for the other three coatings, the values were 1.715 × 10^5^ Ω cm^2^, 1.710 × 10^4^ Ω cm^2^, and 598 Ω cm^2^, respectively. It was generally accepted that a higher low-frequency impedance modulus indicates a better anti-corrosion performance. Thus, it could be concluded that C4 can effectively protect the metal substrate at the initial corrosion stage, and no erosive ions reach the metal substrate at this time. The high-frequency phase shift reflected on the Bode pattern shows that the slope of the high-frequency region curve is small and close to being parallel to the X-axis, indicating that the phase end point has not been reached at this time. It can be found that the low-frequency impedance modulus of C3 decreased rapidly in Figure 8b, indicating that C3 was greatly affected by the acidic medium. However, when the coatings were soaked for 21 days, the value of C3 changed little, and was always better than those of C2 and C1. C4 had highest impedance modulus in all four coatings. When immersed for 31 days, the low-frequency impedance modulus of the C4 decreased to 5.716 × 10^4^ Ω cm^2^, and the metal substrate was corroded. The low-frequency impedance modulus of C3 and C2 for 31 days was 1/5 and 1/10 of that of C4, while the low-frequency impedance modulus of C1 was extremely low, and the coating had long since failed. In contrast, C4 had the best resistance and a certain anticorrosion ability in the acidic medium.

### 3.3. Tafel Curve Analysis

**Figure 9:**
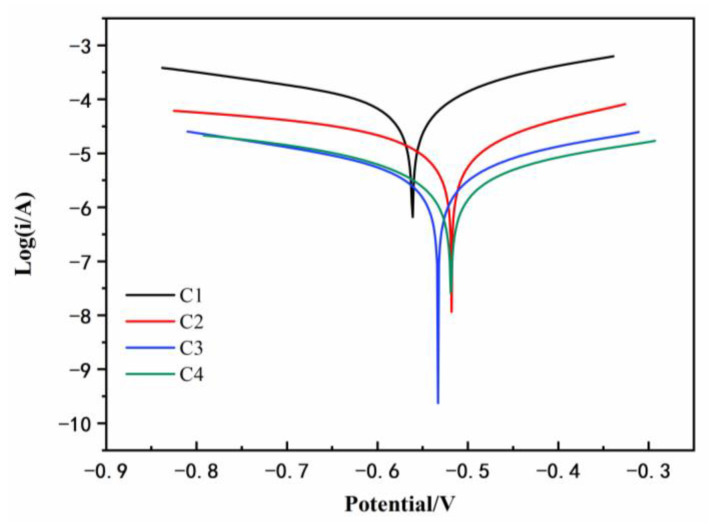
Tafel curves for C1, C2, C3, and C4 after immersion for 31 d.

Figure 9 shows the Tafel curve of carbon steel coated with C1, C2, C3, and C4 immersed in the simulated acid rain solution for 31 d. The characteristic parameters derived from the curves, including the corrosion potential E_corr_, corrosion current density I_corr_, polarization resistance R_p_, and coating protection efficiency η of four coatings in simulated acid rain solution after immersion for 31 d, are listed in Table 3 for better comparison. Though a slight difference in the corrosion potential among the four coatings can be observed, C4 had the smallest corrosion current density, which was 1.526 × 10^−6^ A/cm^2^, and the polarization resistance was 2.305 × 10^4^ ohm. Compared with C1 in the control group, the corrosion current density decreased by nearly two orders of magnitude, and the polarization resistance was two orders of magnitude higher. This revealed that that the anticorrosion performance of C4 was significantly improved after immersion for 31 days. The corrosion current density of C3 was 8.510 × 10^−6^ A/cm^2^ and the polarization resistance was 1.112 × 10^4^ ohm. Compared with C4, the two were in the same order of magnitude, but there was still a certain gap. It can be attributed to the nano-filling effect provided by the CeO_2_ particles. However, the granular shape of CeO_2_ has a limited blocking effect on its path during the infiltration process of water molecules. The hydrolysis of CeO_2_ particles is weakened under acidic conditions, and Ce(OH)_4_ cannot be stably generated, resulting in a declined anticorrosion performance in the acidic medium. The corrosion current density of C2 was 1.328 × 10^−6^ A/cm^2^, which was in the same order of magnitude as that of C1. This is owing to the poor compatibility between GO and EP, and the serious stacking phenomenon of the lamellae meant the prepared coating cannot effectively protect the metal base. Then, the protection efficiency of the coating was judged by calculating the reduction of the corrosion current density of each coating compared with C1. The EP coating performed poorly in acidic solutions. Among the four coatings, the protection efficiency of C4 was the highest, which was 98.62%, indicating that adding CeO_2_–GO (4:1) nanocomposite to EP can significantly improve the protection efficiency of the coating in the simulated acid rain solution.

### 3.4. Mott–Schottky Curve Analysis

**Figure 10:**
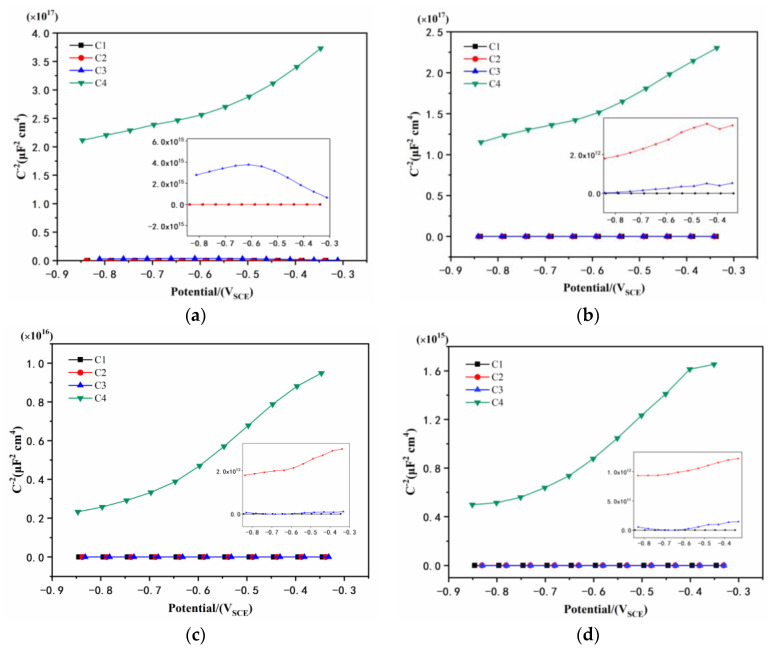
Mott–Schottky curves: (**a**) 1 d, (**b**) 11 d, (**c**) 21 d, and (**d**) 31 d.

Figure 10 shows the Mott–Schottky curve of carbon steel coated with different coatings immersed in the simulated acid rain solution. The slope of the Mott–Schottky curve of C4 was the largest, and the carrier density was the smallest when soaked for 1 day [41]. Basically, a smaller number of carriers migrating inside the coating indicates a better corrosion resistance. The second largest slope of the Mott–Schottky curve was C3, and its slope had both positive and negative values, meaning n-type and p-type semiconductor recombination [42]. The potential barriers caused by different semiconductor types make the migration of carriers difficult. Therefore, the carrier density of C2 and C1 was small. With the prolongation of soaking time for 11 days and 21 days, C4 still had the largest slope. However, the slope of C2 was larger than that of C3, indicating the poor long-term performance of C3. Although the slope of C2 exceeds that of C3, the carrier density of C2 was 1.678 × 1019 cm^−3^, while the carrier density of C4 was only 3.495 × 1014 cm^−3^ after immersion for 31 days, widening the gap with the other three coatings. CeO_2_ particles do not hydrolyze and generate volume-expanded Ce(OH)_4_ in a neutral or alkaline environment, but the presence of CeO_2_ particles in GO causes its lamellae to stretch and not stack up. Compared with other coatings, it can better resist the intrusion of acidic media and show relatively good protective performance.

## 4. Corrosion Morphology Comparison

### 4.1. Corrosion Depth Analysis of Substrate

**Figure 11:**
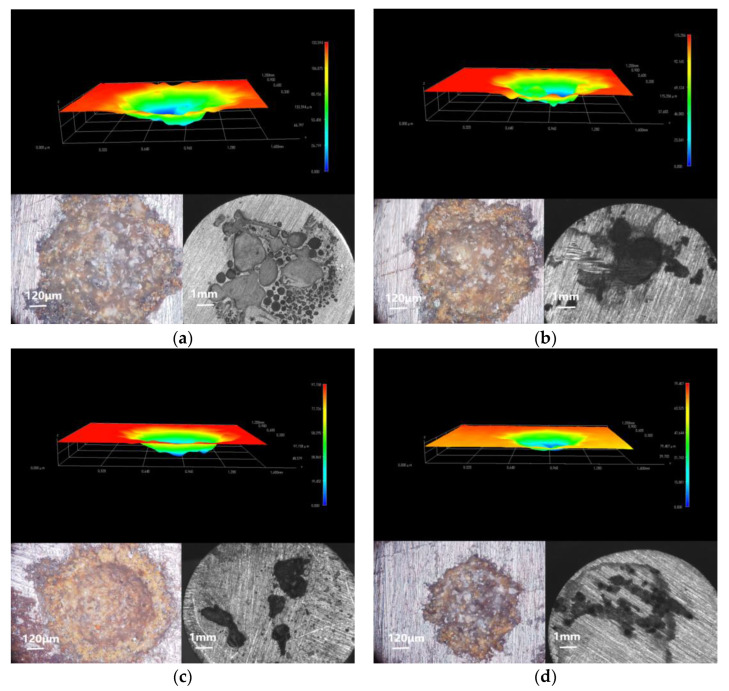
Three-dimensional stereo microscope image, 3D fitted morphology, and stereo microscope image of metal substrate immersed for 31 d: (**a**) C1; (**b**) C2; (**c**) C3; and (**d**) C4.

According to the above electrochemical tests, when soaked for 31 days, all of the coatings suffered a certain degree of corrosion, and the corrosion of the EP coating in the control group was serious. Three-dimensional stereo microscope images, 3D fitted morphology, and stereo microscope images of metal substrate coated by EP, GO, CeO_2_, and CeO_2_–GO (4:1) are shown in Figure 11. Three-dimensional stereo microscope images can show the depth of erosion, while 3D fitted morphology and stereo microscope images show the morphology of the corrosion area. It can be seen from Figure 11a that the corrosion depth of C1, C2, C3, and C4 substrate was 133.594 μm, 115.206 μm, 97.158 μm, and 79.407 μm, respectively. The corrosion depth of C4 was the smallest compared with the other three coatings. There were few areas of connection corrosion pits in Figure 11d. Only the accumulation of corrosion products on the surface was connected, and there was no large-area connection phenomenon. The diameter of the corrosion pit was the smallest, indicating that the coating had a good anti-corrosion effect and can effectively delay the damage of the corrosive medium to the substrate, thereby delaying the corrosion of the metal. However, the accumulation of corrosion products on the EP-coated metal substrate did not significantly slow down the corrosion rate, which was owing to the large number microbubbles caused by the continuous volatilization of the solvent and the air brought into the process of the stirring and curing of the epoxy resin. When the corrosive medium invades the coating, the existence of internal pores and micro-cracks makes the corrosive medium unimpeded. C1 has a weak protection ability and cannot effectively prevent the erosive medium from invading the metal substrate. There was a huge area of corrosion on the metal substrate with large corrosion depth in C2 and C3. Owing to the poor compatibility of GO with epoxy resin and GO itself, which is easy to agglomerate and stack, it cannot exhibit shielding and barrier properties as a two-dimensional material in EP coatings. Compared with the above electrochemical tests, it can be seen that the protective effect of the C3 on the substrate decreases significantly with the prolongation of the soaking time, and the shielding effect on the erosive ions lacks long-term property. With the continuous intrusion of corrosive medium, the ability of nano CeO_2_ particles to adsorb and release oxygen cannot resist excessive oxidation, so its later corrosion resistance is insufficient.

**Figure 12:**
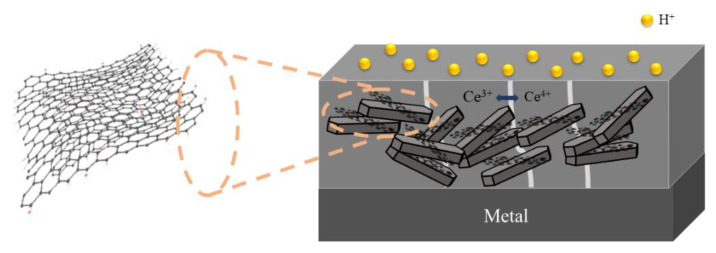
CeO_2_–GO/EP coating under acidic medium.

The resistance of C4 to acidic corrosive medium is poorer than that of saline-alkali, and both the corrosion area and the corrosion depth become larger [43]. On the one hand, owing to the strong penetration of the EP coating by the acidic medium, the epoxy resin has poor protection ability. On the other hand, the hydrolysis volume of CeO_2_ in an alkaline environment expands and blocks the transport path of the erosive medium. In an acidic environment, the hydrolysis products of CeO_2_ particles cannot exist stably, and H+ constantly breaks its hydrolysis balance and penetrates into the metal substrate through the gaps between the particles. Figure 12 shows the mechanism of the CeO_2_–GO/EP coating under acidic medium. When comparing the anti-corrosion performance of prepared coatings subjected to the acidic medium or neutral/alkaline medium, the radius difference between H^+^ and Cl^−^ should be noted. The inherent porous microstructure of the EP provides a quick channel for H^+^ to move fast within the coating, and leads to the inferior anti-corrosion performance of the EP coating subjected to the acidic medium.

### 4.2. Corrosion Area Analysis of Substrate

**Figure 13:**
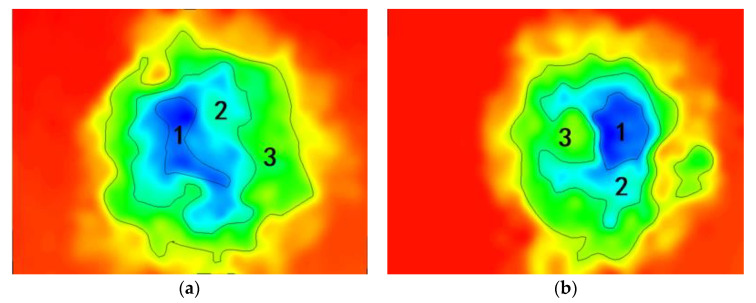

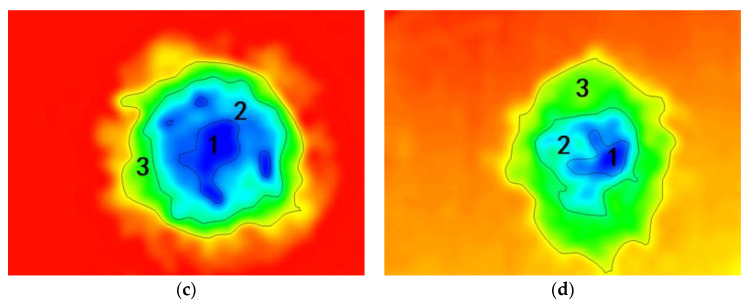
Body microscopic corrosion analysis of metal substrate immersed for 31 d: (**a**) C1; (**b**) C2; (**c**) C3; and (**d**) C4.

Figure 13 shows the body microscopic corrosion analysis of each coating’s substrate immersed for 31 d. Table 4 and Table 5 shows the result of the analysis of the corresponding corrosion areas in Figure 13. Combined with Table 4 and Table 5 and Figure 13, it can be seen that the total corrosion area of C4 was 4578 μm^2^, which was the smallest among the four groups of coatings. At the same time, observing area 1, the deepest area of corrosion, shows that the area of C4 was the smallest, which was 328 μm^2^. This indicated the corrosion medium caused the substrate to corrode and reached the smallest depth. Area 3 accounts for the largest area of C4 corrosion area, indicating that the accumulation of corrosion products on the surface of the substrate delays the continued development of the erosive medium, and it is difficult for water and other erosive ions to enter the deeper substrate. The relationship between the total corrosion area and the deepest corrosion area is C1 > C2 > C3 > C4. It can be seen that C3 has a certain protective effect on the metal substrate, and its total corrosion area is comparable to that of C4, while the protective effect of C2 on the substrate is poor, but better than that of C1. The lamellar structure of GO is stretched by CeO_2_, which improves the interception efficiency of erosive media. At the same time, the hydrolysis of CeO_2_ particles blocks the transmission channel of the corrosive medium inside the coating. So, even if some corrosion occurs on the metal substrate, the subsequent accumulation of corrosion products and the blockage of the transmission channel will greatly reduce the development speed of corrosion.

## 5. Conclusions

In this paper, the effect of the CeO_2_–GO (4:1) nanocomposite on the anticorrosion properties of epoxy coating in simulated acid rain solution were investigated using open circuit potential, electrochemical alternating current impedance spectroscopy, Mott–Schottky curve, and Tafel curve. These techniques concur in pointing out that the CeO_2_–GO (4:1) nanocomposite coating on Q235 carbon steel improves the resistance to corrosion in a simulated acid rain solution. The following conclusions can be reached based on experimental evidence.

(1) The CeO_2_–GO (4:1)/EP coating induced a higher positive corrosion potential (E_corr_) and lower corrosion current density (I_corr_) than those of the EP coating, GO/EP coating, and CeO_2_ coating in a simulated acid rain solution. The coating protection efficiency was as high as 98.62%.
(2) The stereomicroscope analysis indicated that the CeO_2_–GO (4:1)/EP coating had the best barrier performance to the erosive medium. At the same time, the accumulation of corrosion products and the good compactness of the coating limited the further penetration of erosive ions and slowed the corrosion rate.
(3) The resistance of the CeO_2_–GO (4:1)/EP coating to acidic corrosive medium was relatively poorer than that of neutral and saline-alkali corrosive medium. On the one hand, epoxy resin had a poor protection ability to the acidic medium. On the other hand, the hydrolysis product of CeO_2_ could not exist stably, and H^+^ constantly broke its hydrolysis balance.

## Figures and Tables

**Table 1:** Basic performance parameters of graphene oxide.

Solid Content (%)	pH	Viscosity (mPa·S)	Mass Fraction of C (%)	Mass Fraction of O (%)	Mass Fraction of Cl (%)
45 ± 5	1.7 ± 0.3	≥2500	47 ± 3	42 ± 4	<1.0

**Table 2:** Composition of Q235 carbon steel.

Component	C	Mn	Si	S	P
Mass fraction	0.15	0.49	0.29	0.042	0.026

**Table 3:** Basic performance parameters of graphene oxide.

Coating	E_corr_ (V)	I_corr_ (A/cm^2^)	η (%)	R_p_ (ohm)
C1	−0.566	9.196 × 10^−5^	-	5.090 × 10^2^
C2	−0.525	1.328 × 10^−5^	85.56	3.345 × 10^3^
C3	−0.561	8.510 × 10^−6^	90.75	1.112 × 10^4^
C4	−0.518	1.265 × 10^−6^	98.62	2.305 × 10^4^

**Table 4:** Carrier density after immersion for 1 d, 11 d, 21 d, and 31 d.

Coating	1 d N_D_ (cm^−3^)	11 d N_D_ (cm^−3^)	21 d N_D_ (cm^−3^)	31 d N_D_ (cm^−3^)
C1	7.507 × 10^21^	1.370 × 10^22^	2.156 × 10^22^	1.678 × 10^22^
C2	2.309 × 10^18^	2.334 × 10^18^	3.525 × 10^18^	1.678 × 10^19^
C3	1.919 × 10^15^	8.775 × 10^18^	5.234 × 10^19^	3.528 × 10^19^
C4	2.997 × 10^13^	3.961 × 10^13^	5.888 × 10^14^	3.495 × 10^14^

**Table 5:** Areas of the corresponding corrosion.

Coating	Corrosion Area/μm^2^			
	1	2	3	Total
C1	666	1382	3596	5644
C2	577	960	3558	5095
C3	521	1367	2810	4698
C4	338	816	3424	4578

## Data Availability

Not applicable.

## References

[1] Nazari M.H., Zhang Y., Mahmoodi A., Xu G., Yu J., Wu J., Shi X. (2022). Nanocomposite organic coatings for corrosion protection of metals: A review of recent advances. Prog. Org. Coat..

[2] Jin H., Wang J., Tian L., Gao M., Zhao J., Ren L. (2022). Recent advances in emerging integrated antifouling and anticorrosion coatings. Mater. Des..

[3] Hu C., Zhou Z., Chen G. (2022). Effects of different types of acid rain on water stability of asphalt pavement. Constr. Build. Mater..

[4] Liu Z., Sin Z., Wei H., Zhang J. (2022). Acid rain reduces soil CO2 emission and promotes soil organic carbon accumulation in association with decreasing the biomass and biological activity of ecosystems: A meta-analysis. Catena.

[5] Chang H., Wang X., Wang Y., Li S., Wang J., Liu J., Feng P. (2022). Influence of low vacuum condition on mechanical performance and microstructure of hardened cement paste at early age. Constr. Build. Mater..

[6] Lyu K., Garboczi E., Gao Y., Miao C., Liu X. (2022). Relationship between fine aggregate size and the air void system of six mortars: I. Air void content and diameter distribution. Cem. Concr. Compos..

[7] An B. (2003). Corrosion Behavior of Typical Metal Materials in Acid Rain/Rainwater Environment. Ph.D. Thesis.

[8] Zhu X., Zhao H., Wang L., Xue Q. (2021). Bioinspired ultrathin graphene nanosheets sandwiched between epoxy layers for high performance of anticorrosion coatings. Chem. Eng. J..

[9] Li B.-H., Wang S.-L., Pal S., So P.B., Chen G.-Y., Huang W.-J., Hsu Y.-L., Kuo S.-Y., Yeh J.M., Lin C.H. (2021). Versatile reactions on hydrophobic functionalization of metal-organic frameworks and anticorrosion application. Microporous Mesoporous Mater..

[10] Kumari S., Saini A., Dhayal V. (2021). Metal oxide based epoxy coatings for corrosion protection of steel. Mater. Today Proc..

[11] Samal S., Cibulková J., Čtvrtlík R., Tomáštík J., Václavek L., Kopeček J., Šittner P. (2021). Tribological Behavior of NiTi Alloy Produced by Spark Plasma Sintering Method. Coatings.

[12] Li X.W., Chen Z.L., Hou H.B., Hao L. (2013). Corrosion behaviour of electroless Ni–P coatings in simulated acid rain. Corros. Eng. Sci. Technol..

[13] Yang L., Xin L., Yu H.Z., Ju Z.Y. (2019). Preparation and Properties of Graphene Anticorrosive Coatings forTransmission Towers. IOP Conf. Ser.—Earth Environ. Sci..

[14] Nagai T., Hiratsuka M., Alanazi A., Nakamori H., Hirakuri K. (2021). Anticorrosion of DLC coating in acid solutions. Appl. Surf. Sci..

[15] Wang J., Yang H., Meng Z., Xie B., Yu X., Su G., Wang L. (2022). Epoxy coating with excellent anticorrosion and pH-responsive performances based on DEAEMA modified mesoporous silica nanomaterials. Colloids Surf. A Physicochem. Eng. Asp..

[16] Wang J., Liu L., Yang M., Wu X., Li S., Zhang W., Zhang H. (2022). Modification effect of graphene oxide on oxidation coating of Ti-3Zr-2Sn-3Mo-25 Nb near-β titanium alloy. J. Alloys Compd..

[17] Guo Y., Xu Z., Wang Q., Zu S., Liu M., Yu Z., Zhang Z., Ren L. (2022). Corrosion resistance and biocompatibility of graphene oxide coating on the surface of the additively manufactured NiTi alloy. Prog. Org. Coat..

[18] Araujo A.F., Ferreira M.V., Felisberto M.D., Sicupira D.C., Santos L.A. (2022). Corrosion resistance of a superelastic NiTi alloy coated with graphene–based coatings. Prog. Org. Coat..

[19] Chunder A., Liu J., Zhai L. (2010). Reduced Graphene Oxide/Poly(3-hexylthiophene) Supramolecular Composites. Macromol. Rapid Commun..

[20] Ryu S., Kwon Y.J., Kim Y., Lee J.U. (2020). Corrosion protection coating of three-dimensional metal structure by electrophoretic deposition of graphene oxide. Mater. Chem. Phys..

[21] Verma R., Sharma S., Mukherjee B., Singh P., Islam A., Keshri A.K. (2022). Microstructural, mechanical and marine water tribological properties of plasma-sprayed graphene nanoplatelets reinforced Al2O3- 40 wt% TiO2 coating. J. Eur. Ceram. Soc..

[22] Fan X., Xia Y., Wu S., Zhang D., Oliver S., Chen X., Lei L., Shi S. (2022). Covalently immobilization of modified graphene oxide with waterborne hydroxyl acrylic resin for anticorrosive reinforcement of its coatings. Prog. Org. Coat..

[23] Geng Y., Zhou P., Li S., Cao J., Zhou Z., Wu Z., Liu A. (2022). Superior corrosion resistance of mild steel coated with graphene oxide modified silane coating in chlorinated simulated concrete solution. Prog. Org. Coat..

[24] Wang Y., Wu M., Lu P., Zhou W., Shi X., Yang K., Miao X. (2021). Mechanical and corrosion resistance of cold sprayed zinc (CSZ) nano composite coating enhanced by SiO2-GO hybrid material. Colloids Surfaces A Physicochem. Eng. Asp..

[25] Meng L., Watson B.W., Qin Y. (2020). Hybrid conjugated polymer/magnetic nanoparticle composite nanofibers through cooperative non-covalent interactions. Nanoscale Adv..

[26] Zhang S., Liu J., Tang M., Zhang X., Wu K. (2021). Role of rare earth elements on the improvement of corrosion resistance of micro-alloyed steels in 3.5 wt.% NaCl solution. J. Mater. Res. Technol..

[27] Cao G., Wang Y.-H., Ding Z.-Y., Yang H.-L., Liu Z.-G., Ouyang J.-H., Wang Y.-M. (2022). Tunable corrosion resistance of rare-earth monosilicate to molten calcia-magnesia-aluminosilicate glass by RE-doping strategy. Corros. Sci..

[28] Wang C., Ma R., Zhou Y., Liu Y., Daniel E.F., Li X., Wang P., Dong J., Ke W. (2021). Effects of rare earth modifying inclusions on the pitting corrosion of 13Cr4Ni martensitic stainless steel. J. Mater. Sci. Technol..

[29] Zhang Q., Xia X., Chen P., Xiao P., Zhou W., Li Y. (2022). Current research art of rare earth compound modified SiC-CMCs for enhanced wet-oxygen corrosion resistance. Ceram. Int..

[30] Cai G., Xiao S., Deng C., Jiang D., Zhang X., Dong Z. (2021). CeO2 grafted carbon nanotube via polydopamine wrapping to enhance corrosion barrier of polyurethane coating. Corros. Sci..

[31] Wu Y., Jiang F., Qiang Y., Zhao W. (2021). Synthesizing a novel fluorinated reduced graphene oxide-CeO2 hybrid nanofiller to achieve highly corrosion protection for waterborne epoxy coatings. Carbon.

[32] Li D., Zhang Z., Cui X., Feng L., Zhang D., Jin G., Liu J., Zheng W. (2022). Effect of graphite/CeO2 on microstructure and tribological property of plasma cladded Co-based coatings. Mater. Chem. Phys..

[33] You S., Jiang C., Wang L., Xing S., Zhan K. (2022). Effect of CeO2 nanoparticles on the microstructure and properties of the NiCo-CeO2 composite coatings. Vacuum.

[34] Li H., Wang J., Yang J., Zhang J., Ding H. (2020). Large CeO2 nanoflakes modified by graphene as barriers in waterborne acrylic coatings and the improved anticorrosion performance. Prog. Org. Coat..

[35] An K., Sui Y., Qing Y., Yang C., Long C., Wang L., Liu C. (2021). Synergistic reinforcement coating with anti-corrosion and UV aging resistance by filling modified CeO2 nanoflakes. Colloids Surf. A Physicochem. Eng. Asp..

[36] Liu X., Jie H., Liu R., Liu Y., Li T., Lyu K. (2021). Research on the Preparation and Anticorrosion Properties of EP/CeO_2_-GO Nanocomposite Coating. Polymers.

[37] Liu X., Liu R., Li T., Liu Y., Liu L., Lyu K., Shah S. (2021). Research on the Anticorrosion Properties of CeO_2_-GO/EP Nanocomposite Coating in Simulated Sea Water. Polymers.

[38] Lyu K., Liu X., Liu R., Yang H., Qiao Y., Shah S.P. (2022). Evaluation of the Anticorrosion Performance of CeO_2_-Modified Graphene Oxide Nanocomposite Epoxy Coating Subjected to Simulated Saline-Alkali Solution. Polymers.

[39] Hanifah M.F.R., Jaafer J., Othman M.H.D., Ismain A.F., Rahman M.A., Yusof N., Salleh W.N.W., Aziz F., Ajid A.Z.A. (2021). Advanced ternary RGO/bimetallic Pt-Pd alloy/CeO2 nanocomposite electrocatalyst by one-step hydrothermal-assisted formic acid reduction reaction for methanol electrooxidation. J. Environ. Chem. Eng..

[40] Araneda A.A.B., Kappes M.A., Rodríguez M.A., Carranza R.M. (2022). Pitting corrosion of Ni-Cr-Fe alloys at open circuit potential in chloride plus thiosulfate solutions. Corros. Sci..

[41] Reddy I.N., Manjunath V., Shim J. (2021). Structural and optical properties, electrochemical impedance spectroscopy, and Mott–Schottky analysis of ZnFe2O4 nanoparticle-decorated V2O5 rectangular nanosheets for photoelectrochemical applications. J. Environ. Chem. Eng..

[42] Fattah-Alhosseini A., Vafaeian S. (2015). Comparison of electrochemical behavior between coarse-grained and fine-grained AISI 430 ferritic stainless steel by Mott–Schottky analysis and EIS measurements. J. Alloys Compd..

[43] Hiromoto S., Itoh S., Doi K., Katayama H., Akashi T. (2022). Short- and long-term corrosion behavior of carbonate apatite-coated Mg-4mass% Y-3mass% RE alloy in cell culture medium. Corros. Sci..

